# Movement ecology of the carnivorous woolly false vampire bat (*Chrotopterus auritus*) in southern Mexico

**DOI:** 10.1371/journal.pone.0220504

**Published:** 2019-07-29

**Authors:** Ivar Vleut, Gerald G. Carter, Rodrigo A. Medellín

**Affiliations:** 1 Instituto de Ecología, Universidad Nacional Autónoma de México, Mexico City, Mexico; 2 Department of Evolution, Ecology and Organismal Biology, The Ohio State University, Columbus, Ohio, United States of America; Kyoto University, JAPAN

## Abstract

Deforestation is a critical threat to bats. The woolly false vampire bat *Chrotopterus auritus* is a carnivorous bat that is both an indicator species for well-conserved forests and a threatened species in Mexico and other countries due to deforestation. We currently lack the information needed to assess the effects of forest fragmentation and destruction on their populations and to develop plans for their conservation. We used GPS loggers to study the movement patterns of *C*. *auritus* in southern Mexico. We observed 72 foraging nights by GPS-tagging 10 individuals from two colonies on 32 occasions in a highly disturbed heterogeneous landscape with extensive deforestation (Hormiguero), and in a more homogeneous, well-preserved forested landscape (Monterrey). Tracked false vampire bats averaged a home range of 108.24 ha, a core foraging area of 3.78 ha and average maximum flight distances of 2.06 km. The bats ranged farther and flew significantly longer distances in Hormiguero than in Monterrey, with males flying longer and more variable distances. They used the well-preserved semi-deciduous forest more often than secondary forest and agricultural fields for traveling and foraging, but the bats occasionally moved and hunted along the borders of secondary forest and agricultural fields adjacent to semi-deciduous conserved forest areas. Although this carnivorous bat might cope with some fragmentation, we suggest that large well-preserved forested areas are highly important for its conservation.

## Introduction

The extraordinarily rapid rate of biodiversity loss marks the start of a sixth extinction event [1,2]. Despite some limited success of efforts to prevent extinctions [3,4], species extinction rates continue to increase [5] largely due to deforestation and habitat fragmentation [6,7]. While some species use fragmented or deforested habitats, others rely on large, well-preserved forested areas and are heavily affected by changes in environmental conditions and community composition caused by deforestation and fragmentation [8–10]. The conservation of these forest-dependent species requires understanding how different species use forested habitats. Detailed information on the movements, home ranges, foraging areas, and habitat preferences of different species are necessary to identify conservation needs, and to predict the effect of deforestation and habitat loss on populations.

The carnivorous woolly false vampire bat *Chrotopterus auritus* has been considered an indicator species for well-preserved forests [9,11,12] although our knowledge of its behavior and ecology is limited. According to the UICN red list, *C*. *auritus* is globally considered to be of least concern [13], and has a wide distribution that extends from southern Mexico to northern Argentina and Paraguay [14]. However, it is listed as threatened and considered rare in Mexico [15] and Argentina [16], and it is considered ‘potentially vulnerable’ in Paraguay [17]. Although not yet globally endangered, deforestation is likely to threaten their populations in the future.

*Chrotopterus auritus* is an opportunistic hunter that feeds mostly on small mammals, large insects, and birds, but it can also feed on fruits, pollen and reptiles and amphibians [18–26]. The restrictions on the diet of *C*. *auritus* are probably influenced by morphological specifications of the species. The relatively large body mass and short broad wings may cause this carnivorous bat species to hunt in cluttered areas, to have relatively small home ranges, and to move short distances from roost to hunting locations where they probably spend a considerable amount of time perched and listening for prey sounds [20]. Most of the reported captures of this species occur in extensive, well-preserved late-successional forests [9,11,27–33], but the bats do not completely avoid more fragmented, degraded forests and even secondary growth forest [34–37]. Gathering information on the movement ecology of *C*. *auritus*, can improve our understanding of its reliance on well-preserved forested habitat and the effect of future forest fragmentation and destruction on their populations.

Miniaturized GPS makes it possible to track the movement of bats with much greater precision and efficiency than radio-tracking [38–39]. We used GPS to track movements and habitat preference of *C*. *auritus* in both a heterogeneous landscape with increased anthropogenic disturbance and a homogeneous well-preserved, forested landscape. We expected bats (i) to move and hunt mostly inside late-successional forests in an unfragmented, well-preserved landscape, but in a more fragmented landscape, we expected them (ii) to move and hunt in secondary forest, (iii) to avoid agricultural fields, (iv) to avoid low dry forest (due to the low forest height and high tree density), and (v) to have larger home ranges and fly farther to hunting grounds in fragmented landscapes in comparison with the well-preserved late-successional forest site.

## Methods

### Study area and land use mapping

The climate in the study area is humid and warm with a mean annual temperature of 26.0°C and a mean annual precipitation of 1350 mm [40]. The rainy season runs from June to October and the dry season from November to May. The predominant vegetation surrounding the two roosts was tropical semi-deciduous forest dominated by *Brosimum alicastrum*, *Manilkara zapota*, *Metopium brownei*, and *Alseis yucatanensis* trees. These trees can reach up to 30 meters, but in our study areas they reached a maximum height of only 15 to 20 meters due to shallow soils causing low water availability [41–43] and hurricane disturbances [44].

Each of the two colonies of *C*. *auritus* we observed occupied one roost, both of them located in the state of Campeche in southeastern Mexico. The first roost (Hormiguero roost) was located inside structure II (18°24'30.73"N, 89°29'26.01"W) of the archeological site of Hormiguero near the community of Eugenio Echeverría Castellot II, just outside of the Calakmul Biosphere Reserve. When we first equipped bats with GPS trackers, there were 8 individuals in the roost (2 adult females, 4 adult males, 1 juvenile female, and 1 juvenile male). The second roost (Monterrey roost) was inside a cave (18°20'4.53"N, 90° 6'5.76"W) located in the state-managed reserve of Balam-Ku and contained 7 individuals (2 adult females, 3 adult males, and 2 juvenile males) at the time when we deployed GPS trackers.

Land-use maps of the study areas were generated using a supervised classification technique on a SPOT-6 satellite 2014 image with four multispectral bands and a spatial resolution of 10 m. We rectified the land-use map using random ground control points and with field surveys where we traced the movement of the tagged *C*. *auritus* individuals. For habitat selection analyses, we classified areas into medium semi-deciduous forest, dry low semi-deciduous forest, secondary forest, agricultural fields, savannah (mixed woodland grassland), aguadas (ponds), and areas of no vegetation cover. The areas of no vegetation cover included areas where the topsoil layer was removed and the rock substrate was exposed (limestone extraction), paved roads, and urban areas. The areas classified as secondary forest were areas of secondary woody growth after human or natural disturbances and were similar in structure and age (10–15 years old). As part of the forest management plan of the area of Hormiguero, land owners decided to abandon large areas of agricultural fields 10 years ago to reforest and conserve them. They entered into a program called “Servicios Ambientales del Bosque (SAB)” from the National Forestry Commission of Mexico (CONAFOR) from which the land owners receive subsidies from the government to conserve forested areas.

### Bat capturing and tracker attachment

We captured and tagged 10 bats with GPS trackers on 5 occasions in July, September, and December of 2016 and February and May of 2017. We were unable to tag bats from the Monterrey roost during July 2016 and May 2017. Bats were captured inside their roosts using a butterfly net or 3 m x 2.4 m mist nets outside the cave. For each bat, we determined sex, age, reproductive condition (non-reproductive, enlarged testicles, pregnant, lactating, post-lactating), body mass (using Pesola spring scales), and forearm length, and then clipped hair to uniquely mark individuals and to place GPS. The GPS trackers were protected with a 1–2 mm thick layer of moldable plastic (ThermoMorph). We equipped adult non-pregnant bats with GiPSy-5 (Technosmart, Rome, Italy) GPS receivers (23mm x 12.5mm x 5mm) with a Perma-Type surgical cement (Perma-Type Company Inc., Plainville, CT, USA). All of the GPS were programmed to get a spatial fix every 30 seconds. A fix is a GPS-based datum with information on the date, time, and quality (signal strength and number of satellites used). To study whether individuals hunt in groups, we attempted to tag as many of the bats from the same roost as possible after capturing. In Hormiguero, we obtained data from two individuals during the same nights in July, four individuals in September, three in December, four in February and four in May. In Monterrey, we obtained data during the same nights from three individuals in September, three in December and two in February.

The GPS trackers were removed 3 to 4 nights after placement with an adhesive remover (Uni-Solve, Smith & Nephew, Inc.) and a liquid spray-on bandage (AluSpray, Neogen Corp., Lexington, KY) was used on wounds, in case there were any. The GPS data were downloaded from the trackers once they were removed from the bats. The individuals were not immediately re-equipped with GPS trackers after the removal of the GPS tracker; we waited at least 7 days before re-equipping bats with GPS trackers. The weight of the GPS trackers varied between 5.2% (minimum) and 8.9% (maximum) of the individual body masses, above the 5% threshold of the body mass of *C*. *auritus*, which is commonly recommended for birds and bats [45].*Chrotopterus auritus* is a gleaner and capable of carrying prey weighing over 40% of its body weight and taking it to the roosting site [20]. Previous studies [38,46,47] tested for an effect on the extra weight of the devices by comparing the body mass of bats when captured and equipped with trackers, to their mass when recaptured a few days later for device removal. To assess if the GPS trackers negatively affected foraging, we also measured their body mass before placing the GPS trackers and after individuals were recaptured and trackers were removed. If the bats were negatively affected by the GPS trackers, we would expect to detect a decrease in mean body mass.

We handled all bats following the guidelines for the use of wild mammals in research by the American Society of Mammalogists [48]. The bats were captured with a Scientific Collector’s permit (Colector Científico de Flora y Fauna) issued by the environmental authority in Mexico (SEMARNAT; permit number SGPA/DGVS/07161/15 to R. Medellin). The Institute of Ecology approved the animal manipulation procedures before the start of the study. Permission was granted from the National Institute of Anthropology and History of Mexico (INAH) to work at the Hormiguero Temple. The cave was situated on community property of the town of Centenario in the state of Campeche. The community issued the permission to study the bats in the cave.

### Data analysis

We used Ranges 9 software (Antrack Ltd, Wareham, UK) [49] to analyze the home range and core foraging area. We constructed 100% minimum convex polygons (MCPs) to determine home ranges for each complete night of movement data per individual. Although several methods are available to estimate the home range of animals [50–53], the use of minimum-linkage estimate cluster polygons seems the most fitting technique when it comes to mobile animals with relatively small foraging area [54]. This technique tends to overestimate home ranges, but using this method facilitates the comparison of habitat ranges among species because this method has been used by other recent studies evaluating movement of bats [39, 55–59].

After plotting the locations from the GPS trackers, we distinguished locations that either followed a clear path from one location to another, or that formed a cluster in a relatively small area. We considered the latter to be areas where bats foraged, perhaps using a sit-and-wait strategy. We estimated the core foraging area per individual by visualizing and manually selecting the locations that formed a cluster area of GPS locations and clearly did not form a path from one location to another. We constructed 100% MCP’s from these clusters to estimate the core foraging area. We estimated the maximum travel distance by calculating the straight distance from roost to the farthest fix. To account for effects of moon brightness and position, we created a moon index that was highest (5) when the moon was brightest and at a position of 90 degrees, and that was lowest (1) with a new moon or when the position of the moon did not reach above the horizon.

We defined an evening trip as starting when an individual left the roost and ending when it returned to the roost. With bat identity as a random effect, we tested for the fixed effects of sex, site, the interaction between sex and site, moon, and season on several responses observed during each trip: the number of fixes (natural log), the maximum distance traveled from the roost, the total time the GPS registered locations outside of the roosts, the home range (natural log +1), and core foraging area (natural log transformed) using general linear mixed models (lme4 package) in R (versions 3.3.1 and 3.4.0, R Development Core Team, 2006). We used natural log transformations when the model’s residual distributions were lognormal. For all model fits, we confirmed that model residuals did not deviate from normality using diagnostic plots and a Shapiro-Wilk test (p>0.05). When an interaction effect was not detected, we removed it and refit the model. Degrees of freedom and p-values were based on the Satterthwaite approximation for denominator degrees of freedom using the lmerTest package [60–61]. To convey precision and effect sizes, we used non-parametric bootstrapping (boot package in R) to estimate 95% confidence intervals around means.

To define the available habitat for *C*. *auritus* for each study site, we used the maximum travel distance obtained from a male *C*. *auritus* in Hormiguero (5.3km) as the radius of a circle around the roosts. To investigate whether the habitats were randomly used by *C*. *auritus*, we used the compositional analysis proposed by Aebischer et al. [62]. Briefly, we tested how the used habitat (home range and core foraging area) was related to the available habitat in the area. The significance of habitat selection was tested using Wilks Lambda, and a ranked matrix was built to indicate which habitat was used significantly more or less than others (1000 permutations); higher ranked habitats were of greater importance [62–64]. Habitat compositional analysis was performed using the ADEHABITAT package [65].

### Code availability

We provide R scripts as a supplement to reproduce all analyses in S1 File.

## Results

### Movement behavior

The roosts were inhabited throughout the year by most of the same individuals and were rarely abandoned or only abandoned for a few days. In this case individuals would move to another roost on the other side of the same Hormiguero temple. During the study, the colony in Hormiguero reached a total of eight individuals twice, but on both occasions, after 6–8 months, one or two individuals would be absent from the group. The number of individuals in the Monterrey roost had a maximum number of seven *C*. *auritus* bats in the beginning but the group size dropped to four individuals at the end of the study.

**Fig 1:**
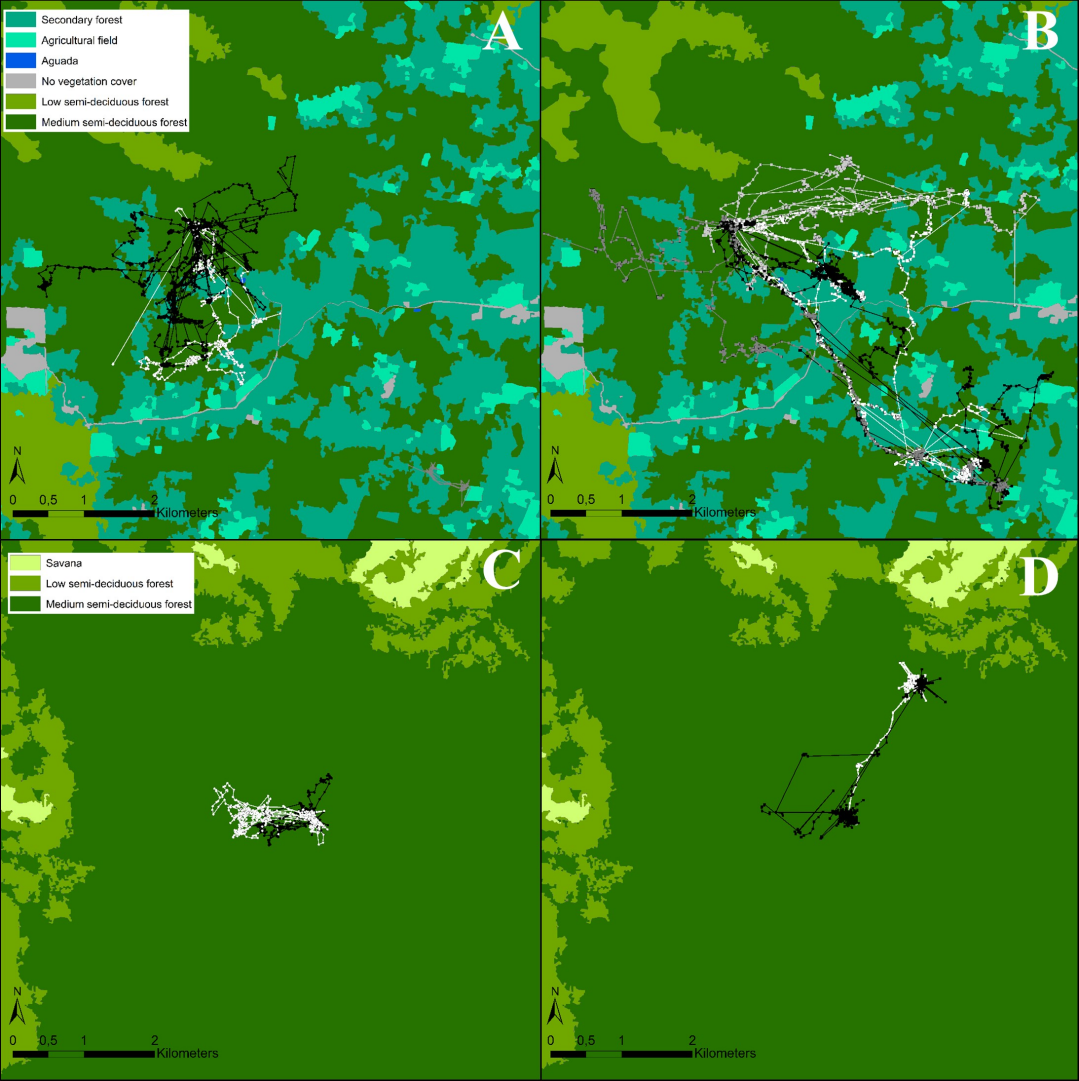
**The total recorded night flight paths of two females (A) and four males (B) in the study site of Hormiguero and two female (C) and two males (D) in the study site of Monterrey.** Different colors depict different individuals.

**Fig 2:**
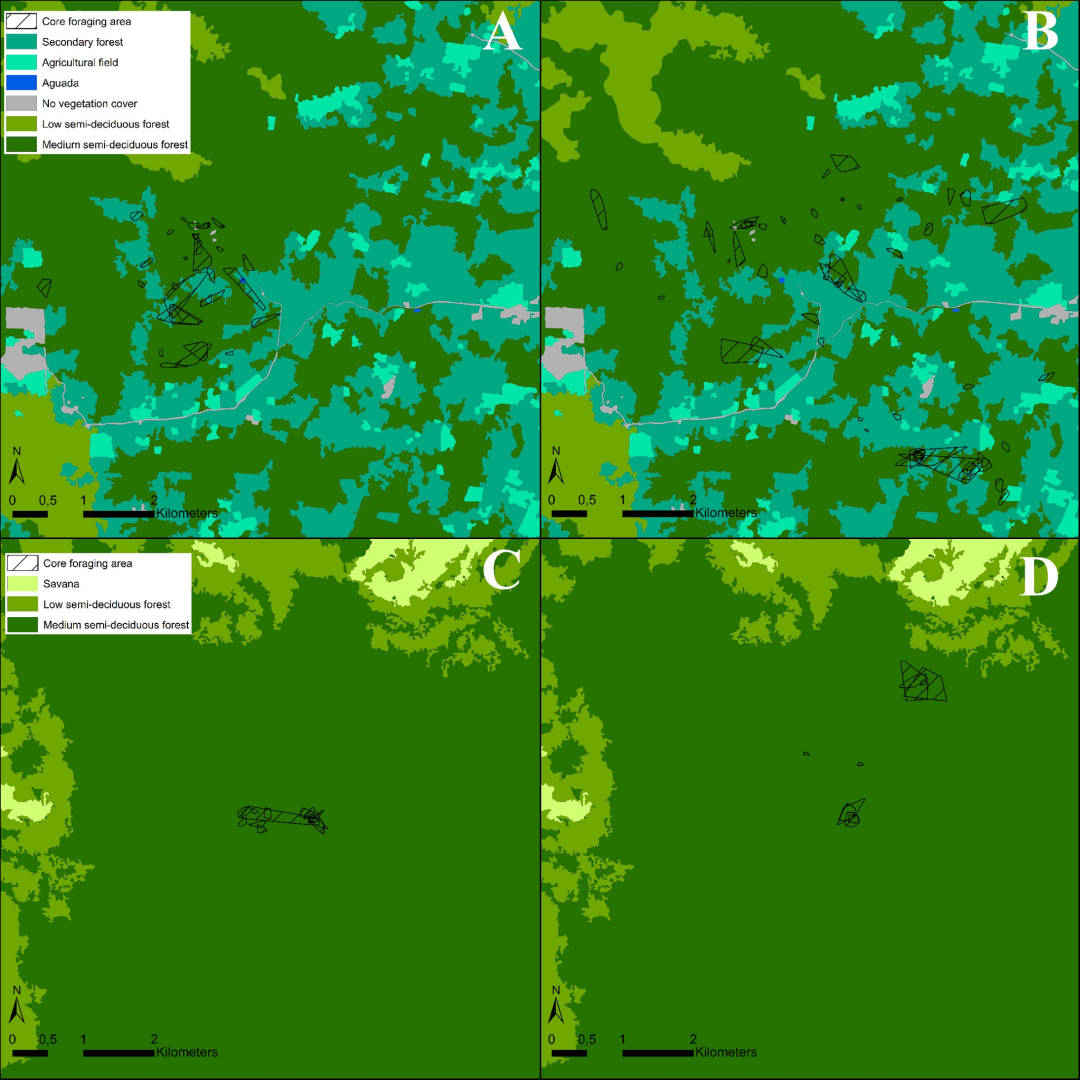
The core foraging ranges per night flight of two females (A) and four males (B) in the study site of Hormiguero and two female (C) and two males (D) in the study site of Monterrey.

**Fig 3:**
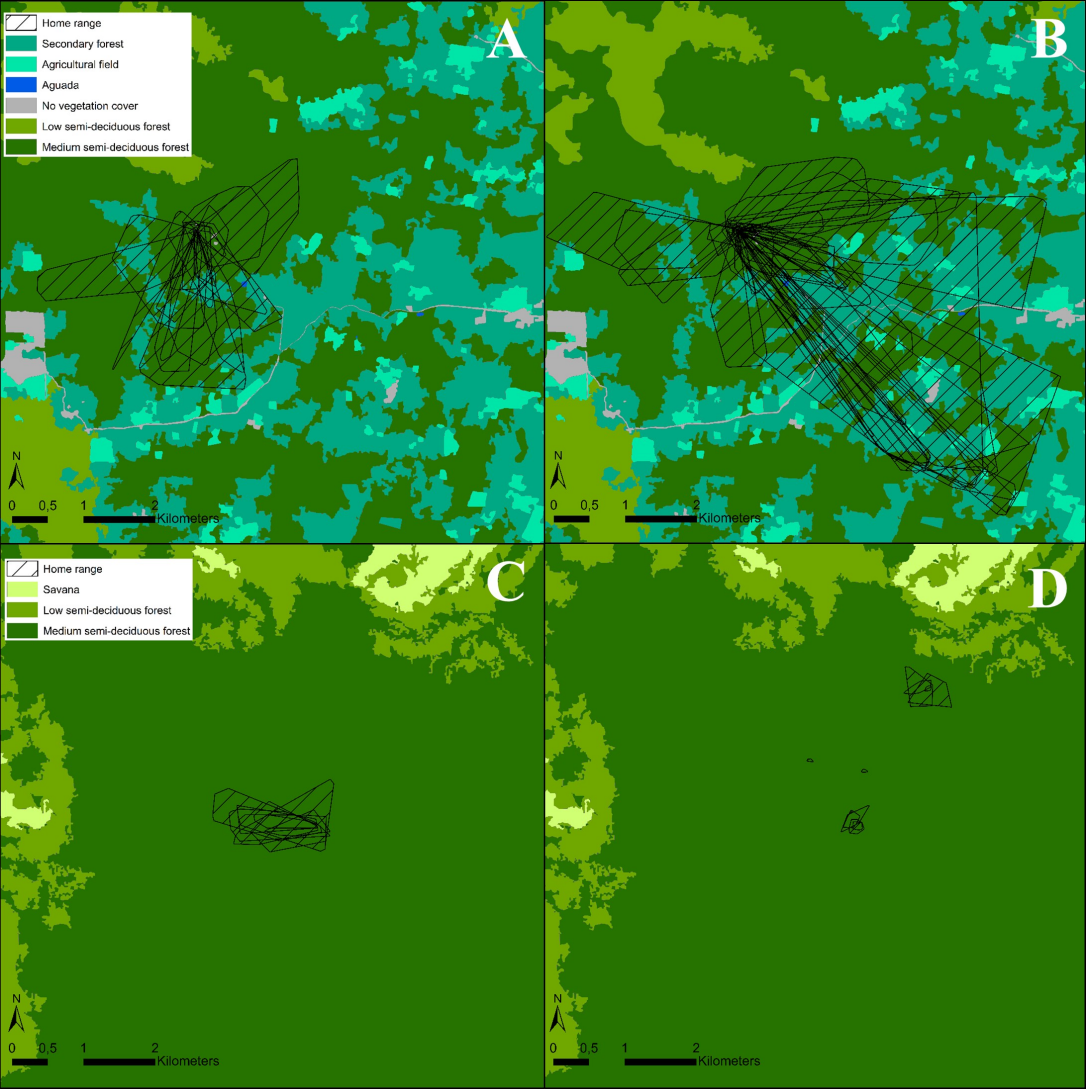
The home ranges per night flight of two females (A) and four males (B) in the study site of Hormiguero and two female (C) and two males (D) in the study site of Monterrey.

We equipped 10 individuals with GPS on 32 occasions and obtained between 1 and 4 nights of movement data per individual, for a total of 17,976 fixes across 72 evening trips (Table 1, S1 Table). The movement of the tagged individuals are shown in Fig 1. The accuracy of the fixes depends on factors such as canopy openness, fix rate and the speed of the animal. The clusters of fixes shown in Fig 1 most likely represent perched individuals, using a sit-and-wait foraging strategy. We identified a total of 30 clusters of fixes (perching events) during 19 nights of tracked data. Individuals spent an average of 172 minutes perched each night. The average duration of a perching event was 126 minutes and the mean number of perching events per night was 1.57. The short distances between clusters of fixes are most likely short flights between perches, while fixes with larger distances between them represent bats in flight. The core foraging area and home range are shown in Fig 2 and Fig 3 respectively.

**Table 1:** Description of the captured and tagged *Chrotopterus auritus* per site and sex.

Ind. #	Sex	Mean FA	Mean mass	Site	Nights of tracked data	Mean number of fixes	Mean time outside of roost	Mean home range (ha)	Mean core foraging range (ha)	Mean distance (km)	Maximum distance (km)
1	Male	83.6	82.6	Hormiguero	10	261.40	03:11:02	213.20	4.94	2.99	4.90
2	Female	84.2	93.8	Hormiguero	13	205.69	05:14:33	66.62	4.66	1.33	2.26
3	Male	82.8	84.4	Hormiguero	10	281.60	06:18:39	185.92	3.49	3.19	5.30
4	Female	84.8	93.7	Hormiguero	5	243.40	05:26:14	68.38	3.98	1.43	2.28
5	Male	81.7	89.0	Hormiguero	9	244.11	05:55:27	108.90	3.46	1.87	4.10
6	Male	81.9	87.0	Hormiguero	8	255.50	04:55:23	113.58	4.61	3.08	5.29
7	Female	81.2	89.0	Monterrey	6	202.50	03:27:12	34.79	3.92	1.12	1.43
8	Male	80.1	77.7	Monterrey	7	205.43	04:54:22	41.33	4.27	1.14	2.44
9	Female	80.7	85.0	Monterrey	2	396.50	08:00:21	39.77	4.15	0.79	0.88
10	Male	79.0	79.0	Monterrey	2	484.00	05:49:31	61.95	9.10	2.33	2.36

Females were larger than males (rank-sum tests; mass: z = 2.286, p = 0.022; forearm: z = 2.027, p = 0.043; Table 1). The mean change in mass of the bats after placement of the GPS (Table 2) showed that the GPS-tagged bats did not tend to lose weight (mean change = +0.32, 95% CI = -2.1 to +2.9 g). The emergence times of bats were ambiguous since the GPS trackers were not always capable of getting a fix just after the bats left their roosts, but the earliest fix we observed was 29 minutes after sunset. The number of fixes averaged 250 per evening trip (95% CI: 208 to 291; n = 72; range 21 to 746) and did not strongly differ between sites (Hormiguero: 95% CI of mean = 197 to 293, range: 21–712, n = 55 trips; Monterrey: 95% CI of mean = 165 to 342, range: 36–746, n = 17 trips) or by sex (95% CI of mean; males: 209 to 315, n = 46 trips; females: 157 to 290, n = 26 trips). We detected no effects of moon (t = 1.1, p = 0.26), or season (t = 1.1, p = 0.27) on the number of fixes, either in the full model or when tested as single effects.

**Table 2:** Mass before and after GPS placements.

Bat ind.	GPS weight (gram)	Proportion of body weight (%)	Body mass before (gram)	Body mass after (gram)	Change (gram)
H1	6	7.7	78	80.5	2.5
H3	6	7.2	83	81.0	-2
H3	6	7.4	81	80.0	-1
H3	5.5	6.9	80	85.0	5
H2	8	8.9	90	91.0	1
H5	6.5	8.1	80	80.5	0.5
H2	6	6.6	91	94.0	3
H1	6	7.3	82	76.0	-6
H7	5.2	6.4	81	75.8	-5.2
M2	6	8.6	70	69.0	-1
M7	7	8.9	79	80.0	1
M5	6	7.1	85	85.0	0
H1	6	7.1	85	88.0	3
H2	5.7	6.3	91	92.3	1.3
M1	6.3	7.7	82	81.7	-0.3
M2	6.38	8.6	74	80.2	6.2
H7	5.85	7.1	82	83.0	1
H2	5.94	6.9	86	86.0	0
H6	6.39	6.7	95	78.6	-16.39
H2	6.39	6	107	93.7	-13.26
H3	5.96	7.4	81	90.1	9.1
H5	6.25	6.5	96	98.8	2.75
H1	6.89	7.3	94	108.1	14.11
M2	6.72	7.6	89	82.3	-6.72
H5	5.44	5.2	105	87.6	-17.44
H7	7.42	8.1	92	99.6	7.58
M1	7.34	7.6	96	92.7	-3.34
H1	5.83	7.9	74	91.0	17
H6	5.61	6.2	90	84.0	-6
H3	6.13	6.3	97	104.0	7
H7	6.1	6.6	93	98.0	5
H6	6.03	7.4	82	84.0	2

The average maximum flight distance traveled was 2.06 km and was influenced by season (t = 3.3, p = 0.0018) and an interaction between site and sex (t = 2.6, p = 0.01). The overall maximum travel distance was roughly twice as great in Hormiguero (mean = 2.4, 95% CI: 2.0 to 2.8 km) compared to Monterrey (mean = 1.2, 95% CI: 0.9 to 1.6 km), but this effect was driven by male movements, which were more variable than female movements (F(45, 25) = 6.95, p < 0.0001). We detected no effects of site (t = 0.67, p = 0.69) or season (t = 0.94, p = 0.36) on distance traveled by females. Males, however, flew farther at Hormiguero than at Monterrey (estimate = 1.92, t = 4.03, p = 0.0002), and male flights were farther in the dry season (estimate = 1.68, 3.52, p = 0.001).

Flight distances did not vary much with moonlight in either females (t = 1.26, p = 0.22) or males (t = 0.24, p = 0.81), but moonlight was the sole predictor of time spent outside the roost (estimate = -0.57, t = -3.43, p = 0.001). Time outside the roost was not predicted by sex (t = 0.51, p = 0.62), site (t = 0.20, p = 0.62), or season (t = 0.81, p = 0.42). The average time outside the roost was 5 hours and 9 minutes. The longest night trip was 10 hours and 8 minutes and the shortest trip was 26 minutes. All of the individuals were back at the main roost after every evening trip, but it is possible that individuals used alternative roosts for part of the night, since the GPS trackers would often lose reception for an extended period of time during the night (max = 4 hours) and would regain satellite reception when a bat was returning to the main roost.

The home range averaged 108.24 ha (SD ± 138.92) and was higher in Hormiguero (mean = 129, 95% CI = 86 to 166 ha) than in Monterrey (mean = 41, 95% CI = 25 to 57 ha; estimate = 0.88, t = 2.73, p = 0.024) with no detected effects of sex (t = 1.22, p = 0.27), moon (t = -1.21, p = 0.23), or season (t = 0.26, p = 0.80) on home range size. The core foraging area averaged 3.78 ha (SD ± 5.14) and we detected no effects of sex (t = 0.06, p = 0.95), site (t = 0.12, p = 0.90), moon (t = 0.21, p = 0.83), or season (t = 1.79, p = 0.08). We never observed multiple tracked individuals visiting the same area at the same time.

### Habitat availability and preference

The main habitat available in both areas was predominantly medium semi-deciduous forest, followed by secondary forest and low semi-deciduous forest in Hormiguero and also predominantly medium semi-deciduous forest followed by low semi-deciduous forest and savannah in Monterrey (Table 3). The comparison between the habitat availability and the home range showed that the habitat use was non-random (λ = 0.0006828, p = 0.001) with medium semi-deciduous forest (80.2%) as the preferred habitat, followed by secondary forest (18.2%), agriculture (0.8%) and finally low semi-deciduous forest (0%). The core foraging area use was also non-random (λ = 0.04067, p = 0.001) and followed a similar preference in habitat as the home range, with medium semi-deciduous forest (79.2%) as the most important habitat. We did not observe the bats moving through low semi-deciduous forest or savannah.

**Table 3:** Percentage of habitat availability assessed for Hormiguero and Monterrey and percentage (+ SD) of home range area and core foraging range within 100% minimum convex polygons (MCP) of *Chrotopterus auritus* tagged individuals.

	Hormiguero			Monterrey		
	% habitat availability	Mean % home range area	Mean % core foraging range	% habitat availability	Mean % home range area	Mean % core foraging range
Medium semi-deciduous forest	59.4	74 (18.2)	75.9 (36.5)	80	100	100
Low semi-deciduous forest	12.2	0	0	16.3	0	0
Secondary forest	20.9	23.87 (17.17)	23.6 (36.8)	0	0	0
Agricultural fields	5.3	1.04 (2.12)	0.44 (3.5)	0	0	0
No vegetation cover	1.8	0.99 (1.22)	0.1 (0.6)	0	0	0
Aguada (pond)	0.4	0.09 (0.18)	0	0	0	0
Savannah	0	0	0	3.8	0	0

## Discussion

This is the first study detailing the movement patterns of the second-largest bat species of the Americas. *Chrotopterus auritus* relied on well-preserved late successional forests; their home range and foraging area were comprised of predominantly well-preserved medium semi-deciduous forest. The size of the home range of *C*. *auritus* differed between the more fragmented Hormiguero compared to Monterrey, and individuals in Hormiguero also flew farther from the roost compared to the better-preserved study areas of Monterrey. Males flew at longer and more variable distances than females.

For tracked *C*. *auritus* bats, the average home range was 108.24 ha, the core foraging area was 3.78 ha, and the average maximum flight distance reached 2.06 km with a longest maximum distance of 5.3 km. Carnivorous bats can be separated from insectivorous and piscivorous bats through a combination of body mass (BM ≥ 0.017 kg), low relative wing loading (RWL; < 36) and low aspect ratio (RA; ≤ 6.3), which makes continuous flights for these species more expensive, but would enhance their maneuverability and allow them to carry high load prey [66]. The largest carnivorous bat, *Vampyrum spectrum* (158 g, AR = 5.4, RWL = 34; [65]), had an estimated foraging area of 3.2 ha during a single tracking event [67]. The frog-eating *Trachops cirrhosus* (44 g, AR = 6.3, RWL = 44; [66]), the smallest of the three carnivorous bats in the Americas, reached a foraging area of 3–12 ha and traveled more than 1.5 km from the roost [68]. *Megaderma lyra* (50 g, AR = 6.2, RWL = 32; [66]), a carnivorous bat species native to Asia, covered distances of 4 km and used foraging areas of 10 ha [69]. Data from the second largest carnivorous bat *Macroderma gigas* (123 g, AR = 6.1, RWL = 34; [66]) showed foraging areas reaching over 60 ha [70] and an average distance traveled of 1.9 km in Australia. Among bats classified as carnivorous from Africa, *Cardioderma cor* (30 g, AR = 5.2, RWL = 31; [66]), presented much smaller feeding areas between 0.1 and 1.01 ha [71] and *Nycteris grandis* (32 g, AR = 5.2, RWL = 35; [66]), used short commuting distances <2.2 km [72]. These carnivorous bat species vary slightly in wing loading and aspect ratio but differ substantially in weight. We lack the data to rigorously link these measures to home range, core foraging area and maximum distance traveled in carnivorous bats. Differences in foraging areas and movements could depend on energetic constraints due to morphological or physiological differences [69,73] or simply landscape differences or seasonal changes in prey abundance [72,74,75].

A fragmented landscape with small well-preserved patches of primary forests could cause bats to fly longer distances and use larger home ranges in the search for appropriate foraging areas. In our study, bats moved farther in the more fragmented landscape of Hormiguero than in the homogenous landscape of well-preserved forests of Monterrey, which is consistent with an effect of habitat, although a replicated sample of sites is necessary to test this hypothesis.

Previous reports indicate that *C*. *auritus* is most likely an opportunistic hunter that feeds mostly on small vertebrates such as rodents, bats, birds, and occasionally insects [19,20,22–26]. Prey commonly taken by *C*. *auritus* include rodent species of the genera *Heteromys* and *Peromyscus* [20], which are commonly found in well-preserved forests, but do not shy away from secondary forest [76]. The distribution of these prey could explain the importance of well-preserved forests for *C*. *auritus*, followed by secondary forest areas. Secondary forests have displayed higher species richness and diversity of small mammals than primary forests [77], but this was most likely related to the size of the secondary forest areas with higher richness and diversity in larger areas of secondary forests [78]. Tagged bats did not seem to forage in the center of secondary forests, but more along the edges. Bats would be unable to perch in agricultural fields due to the lack of trees, but the mosaic of edges of agricultural fields, secondary forests and medium semi-deciduous forest potentially creates areas of high species diversity and an increase in potential prey [79,80]. Similar behavior was documented by Vehrencamp et al. [67], where a radio-tagged *V*. *spectrum* was found foraging in broken woodland, secondary forest, and forest. However, it is yet unclear which prey species are potentially found more often in these edges. Homogenous continuous forests are likely to provide less variety in habitats and possibly prey items, while transition areas between secondary forests, agricultural fields and well-preserved forests could increase the structural and compositional diversity of vegetation, attracting small mammals by providing climbing and foraging substrates and nesting possibilities for birds [81, 82]. On the other hand, small mammals and birds experience an overall increase in predation risk in natural edges [83]. Bats completely avoided low semi-deciduous forest, which are distinguished by their relative low tree height and high tree and understory density [40,42]. More detailed information on the ecology of the prey of *C*. *auritus* is necessary to assess the possible role of prey abundance on *C*. *auritus* movements.

The formation of clusters of fixes in a relatively small area indicates that bats are perched at a certain point and can remain perched for several hours. *C*. *auritus* probably uses a sit-and-wait foraging strategy, but is likely to combine this tactic with short continuous flights. A sit-and-wait strategy can significantly decrease energy consumption by decreasing flight time. The highest energy consumption these bats face is probably commuting between roosts and foraging areas, and attacking and carrying prey [73]. Female bats experience higher energetic costs during reproductive periods [84–86]. We decided not to track pregnant females or females carrying pups, but only deploy GPS units on non-reproductive females or females with volant young. We therefore do not know how female reproductive stage influences foraging behavior, and we can only speculate on the reason why males used foraging areas further away from the roost than females. Sex differences in foraging might contribute to sexual dimorphism in body size [87,88]. *Chrotopterus* females were larger, perhaps to support heavier loads carried during pregnancy [89,90].

We saw no evidence for group foraging. Group foraging in bats might occur when resources are patchy in distribution but seasonally abundant [91]; however, the individuals we tracked never coincided with other tracked bats in space and time, which is consistent with the hypothesis that *C*. *auritus* is a solitary forager that does not hunt in groups.

Our data are also consistent with the hypothesis that an increase in fragmentation of the landscape could increase the distance *C*. *auritus* must move through the landscape to find adequate foraging areas. Preserved primary forests appear to be important for both commuting and hunting; however, contrary to our expectations, the bats would sporadically fly through secondary forests in the fragmented Hormiguero site. Further studies at more sites are needed to understand how fragmentation of well-preserved forests might alter movement and food availability.

Inventory data of Neotropical bat assemblages are often used for assessing biodiversity and ecosystem functioning. With relatively simple methods, such as mist nets and bat detectors, species are categorized into groups according to their responses towards disturbance or land management in a specific area. These studies are important to get a general idea on the local biodiversity and how bat assemblages are shaped and structured by anthropic activities and land use changes, but for species-specific information, studies that include GPS and/or radio-tracking individuals are also important and recommended for monitoring the movement ecology of these mobile animals.

## Supporting information

S1 File R scripts to reproduce all analyses. (R)

S1 Table *Chrotopterus* tracking data. Detailed information per tracked individual.
(CSV)
